# The prediction of fluctuation in the order-driven financial market

**DOI:** 10.1371/journal.pone.0259598

**Published:** 2021-11-18

**Authors:** Fabin Shi, Xiao-Qian Sun, Jinhua Gao, Zidong Wang, Hua-Wei Shen, Xue-Qi Cheng

**Affiliations:** Data Intelligence System Research Center, Institute of computing technology, Chinese Academy of Sciences, Beijing, Haidian, China; Newcastle University, UNITED KINGDOM

## Abstract

Risk prediction is one of the important issues that draws much attention from academia and industry. And the fluctuation—absolute value of the change of price, is one of the indexes of risk. In this paper, we focus on the relationship between fluctuation and order volume. Based on the observation that the price would move when the volume of order changes, the prediction of price fluctuation can be converted into the prediction of order volume. Modelling the trader’s behaviours—order placement and order cancellation, we propose an order-based fluctuation prediction model. And our model outperforms better than baseline in OKCoin and BTC-e datasets.

## Introduction

Risk prediction is one of the important issues that draws much attention from academia and industry. It is the key element for financial decision making and plays an increasing role in the financial market. The fluctuation—the absolute value of the change of price, which is one of the indexes of risk. In this paper, we focus on the fluctuation prediction.

In finance, the traditional methods applied to predict the market include fundamental analysis and technical analysis. Fundamental analysis involves assessing a firm’s equity value based on the analysis of related economic and financial factors [1]. Fundamental researchers study everything that can affect the security’s value, from microeconomic factors like the effectiveness of the company’s management to macroeconomic factors such as the state of the economy and industry conditions. Unlike fundamental analysis, technical analysis is an analysis methodology for forecasting the direction of prices by analyzing statistical trends gathered from trading activities, such as price movements and volumes. It focuses on patterns of price movements, trading signals, and various other analytical charting tools to evaluate a security’s strength or weakness.

Besides, the financial time series models expressed by financial theories have provided classic forecasting methods. For the stationary time series, Yule [2] proposed the autoregressive model(AR) which assumes that the current state is linear to the previous state. Similarly, Walker [3] proposed the moving average model(MA). The difference is that MA concerns the variance. The automatic regression moving average model(ARMA) is a mixed model combined with AR and MA, which combines the advantages of AR and MA. As for the non-stationary time series, the autoregressive conditional heteroscedasticity model(ARCH) adopted by Engle [4] is commonly used, which solves the problem brought by the hypothesis that the variance is constant. In ARCH, the unconditional variance of the model is a constant, but its conditional variance changes with time. Besides, there are many ways to improve [5–7], such as the generalized autoregressive conditional heteroscedasticity model(GARCH) [8], asymmetric power autoregressive conditional heteroscedasticity model(APGARCH) [9].

In recent years, with the development of artificial intelligence, many researchers have applied machine learning methods to the financial field [10–20], including genetic algorithm, convolutional neural network, and support vector machine. Hammad [21] found the artificial neural network has a strong predictive ability and high precision compared with econometric analysis methods. Besides, the researchers attempted to integrate artificial neural networks, moving average autoregressive models, maximum entropy models, particle swarm optimization algorithms, and genetic algorithms into a mixed model [22–26]. Hassan [27] combined the hidden Markov model, artificial neural networks, and genetic algorithms to predict the price of three stocks. Their results show that their methods have significantly improved over other models in accuracy.

However, these models ignore the relationship between order and price. There exist many studies that are concerned with this issue in theory [28–36]. Shi [37] found the change of price is inverse of the volume of best ask and best bid. Yura [38] found that the price change is a proportion of the change of order between ask orders and bid orders. All of these suggest that the order is key for the fluctuation prediction which can not be neglected. In this paper, we focus on the fluctuation prediction using order book data. We find that the volume of the order would change when the price has moved. Based on this observation, the prediction of price change can be converted into the prediction of order volume. Modelling the trader’s behaviour—order placement and order cancellation, we conduct an order-based fluctuation prediction model. Our experimental results show that the proposed model outperforms other methods on OKCoin and BTC-e datasets.

## Results

### Data

We collect order book data from the Bitcoin transaction platform. Compared with the stock market or futures market, it is easy to collect order book data from the Bitcoin market. We use the API provided by the Bitcoin platforms to collect order book data. It includes

- OKCoin was the largest Bitcoin exchange platform in China which consisted of millions of users and billions of turnovers per day. We collected order book data at OKCoin from Nov. 3rd, 2016 to Jul. 28th, 2017.
- BTC-e is one of the largest Bitcoin trading platforms headquartered in Russia, whose data was collected from May 3rd, 2017 to Jul. 26th, 2017.

The data description is in Table 1.

**Table 1:** The basis information about the OKCoin and BTC-e dataset.

Platform	date	country	record
OKCoin	Nov. 3rd, 2016–Jul. 28th, 2017	China	3.8 × 10^6^
BTC-e	May 3rd, 2017–Jul. 26th, 2017	Russia	3.7 × 10^6^

### Price fluctuation

The most widely accepted models state that the variation of share prices is a random process. Investigating the time series of fluctuation on varying time scales |Δ*t*| is useful in probing the underlying nature of the stochastic process. The fluctuation |Δ*Z*(*t*)| are defined as the difference between two successive price X(t).

$$\begin{array}{c} \begin{array}{c} \left|\Delta Z(t)|=|X(t+|\Delta t|)-X(t)\right| \end{array} \end{array}$$ (1)

**Fig 1:**
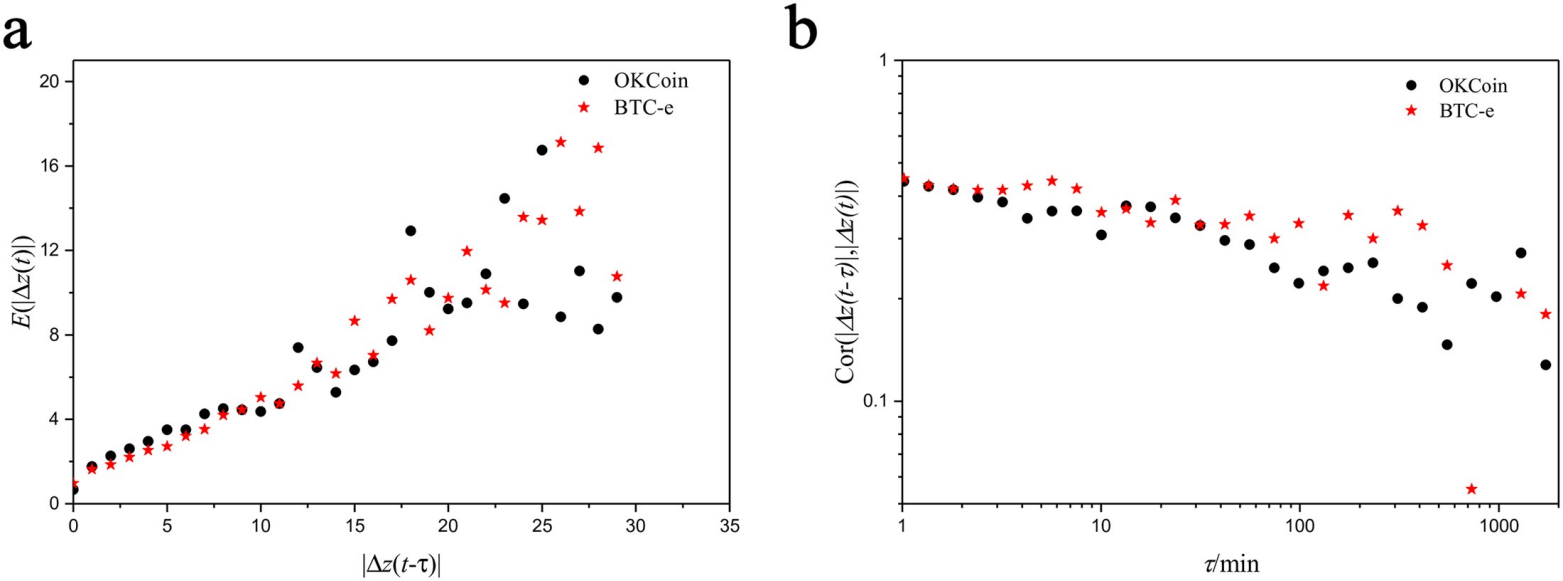
The predictability of the fluctuation in price in Bitcoin transaction platform. **a**. The relationship between the fluctuation of price in time *t* − *τ* and the fluctuation in time *t*. **b**. The autocorrelation of the price fluctuation *Cor*(|Δ*z*(*t* − *τ*)|, |Δ*z*(*t*)|) versus time interval *τ*.

The expected value of fluctuation *E*(|Δ*z*(*t*)|) = ∑_*d*_ *P*(|Δ*z*(*t*)| = *d*)*d*. Fig 1a shows the relationship between the expected value of fluctuation of price at time *t* − *τ* and the fluctuation at time *t*. In Fig 1a, we find that the expected value of fluctuation *E*(|Δ*z*(*t*)|) increases as the fluctuation |Δ*z*(*t* − *τ*)| increases, where it is approximately linear. It suggests that if the fluctuation at time *t* − *τ* is large, the expected fluctuation would be large next time. From this aspect, the fluctuation of price has a strong correlation. Moreover, we measure the autocorrelation of the series of price fluctuation. The autocorrelation is calculated as

$$\begin{array}{c} \begin{array}{cc} & Cor(|\Delta z(t-\tau )|,|\Delta z(t)|)\\ = & \frac{\left\langle [|\Delta z(t)|-\langle |\Delta z(t)|\rangle ][|\Delta z(t-\tau )|-\langle |\Delta z(t)|\rangle ]\right\rangle }{\sigma^{2}\left(|\Delta z\left(t\right)|\right)}, \end{array} \end{array}$$ (2)

while 〈|Δ*z*(*t*)|〉, *σ*(|Δ*z*(*t*)|) are the mean and the standard derivation of |Δ*z*(*t*)|, respectively. In Fig 1b, we find there exists autocorrelation between the fluctuation of price when the time interval *τ* < 10*min*. Besides, the autocorrelation decreases as time interval *τ* increases. When the time interval *τ* = 1 min, the autocorrelation *Cor*(|Δ*z*(*t* − *τ*)|, |Δ*z*(*t*)|) ≈ 0.5; and when *τ* = 10 min, the autocorrelation *Cor*(|Δ*z*(*t* − *τ*)|, |Δ*z*(*t*)|) ≈ 0.2.

### Experiment

In this section, we evaluate the effectiveness of our model in two dataset and compare our model with several state-of-the-art methods for fluctuation prediction. we divide the entire data into two parts. 80% of the data is used for in-sample training, which is used to determine the specifications of the model. And the other is considered as test data. For training, we use mean-square error to optimize our model.

#### Evaluation

To test the prediction of our model, we use the mean-square error and coefficient of determination to evaluate.

- MSE(mean-square error): The mean-square error is the average squared difference between the estimated values and the actual value. It is defined as
  $$\begin{array}{c} MSE=\sum_{t}{\left(\hat{z(t)}-z\left(t\right)\right)}^{2}, \end{array}$$ (3)
  while $\hat{z(t)},z\left(t\right)$ are estimated values and the actual value, respectively.
- *R*^2^ (coefficient of determination): The coefficient of determination is the proportion of the variance in the dependent variable that is predictable from the independent variable(s). The coefficient of determination is defined as
  $$\begin{array}{c} R^{2}=1-\frac{\sum_{t}{\left(\hat{z(t)}-\bar{z}\right)}^{2}}{\sum_{t}{\left(z\left(t\right)-\bar{z}\right)}^{2}}, \end{array}$$ (4)
  while $\hat{z(t)},z\left(t\right)$ are estimated values and the actual value, respectively. And $\bar{z}$ is the average of the actual value.

#### Baseline

We compare our model with several commonly used methods, which are briefly described below.

- ARMA: ARMA(Autoregressive moving average) is one of the common methods for financial time series, which is a mixture of autoregressive model and moving average model. In this paper, we use the change of price series as input data, and apply the ARMA method for fluctuation prediction. The autoregressive polynomial degree and moving average polynomial degree are set into 10.
- SVR: The SVR(support vector regression) method is widely used for the stock prediction in the previous work [39–41]. In this paper, we use the change of price series as input data and apply the SVR method for fluctuation prediction.
- ANN: ANN(Artificial Neural Network) is one of the commonly used machine learning methods. There is much previous work that has been reported to used ANN to predict stock price [42, 43]. In this paper, we use the change of price series as input data and apply the ANN method for fluctuation prediction. The ANN model consists of three layers with 20 nodes. And two Relu are used in the first two layers.
- CNN: CNN(Convolutional Neural Network) is a class of deep neural networks, which is good at capturing the local features. Persio [44] used CNN to predict stock price. In this paper, we use the change of price series as input data and apply the CNN method for fluctuation prediction. The convolutional neural network consists of two convolutional layers. The parameters of these two convolutional layers are (kernel = 1×3, filters = 4, stride = 1, active function = ReLU) and (kernel = 1× 3, filters = 8, stride = 1, active function = ReLU). And two max-pooling layers are used after convolutional layers respectively. The bottom of the network is built by two fully connected layers.

#### Experiment result

Table 2 shows the experiment result in OKCoin dataset. The MSE material is 0.761, 0.780, 0.749, 0.783 for four baseline methods when time interval Δ*t* = 10*s*. SVR performs the worst in MSE material. It is because the complexity of SVR is much higher than others when the train dataset is larger. Thus, the experiment result of SVR does not work well when the train time is limited. ANN and CNN perform better than SVR. And the performance of ARMA is the best in four baseline methods. However, the MSE metric in our model is 0.712. It is approximately 5% improvement compared with ARMA, which is the best method in the baseline. Similarly, our method outperforms when Δ*t* = 20*s*, 40*s*, 80*s*. Table 3 shows the experiment results in the BTC-e dataset. And we find a similar experiment results in the BTC-e dataset except the experiment result when *τ* = 80*s*. In summary, our method performs better than the baseline for MSE material.

**Table 2:** The experiment result in OKCoin dataset.

		ANN	CNN	ARMA	SVR	Our model
*τ* = 10*s*	MSE	0.761	0.780	0.749	0.783	**0.712**
	*R* ^2^	0.164	0.143	0.174	0.140	**0.219**
*τ* = 20*s*	MSE	1.625	1.652	1.577	1.657	**1.531**
	*R* ^2^	0.147	0.132	0.172	0.130	**0.196**
*τ* = 40*s*	MSE	3.622	3.674	3.589	3.667	**3.398**
	*R* ^2^	0.0981	0.0852	0.106	0.0871	**0.154**
*τ* = 80*s*	MSE	7.437	7.257	6.944	7.353	**6.887**
	*R* ^2^	0.0397	0.0630	0.103	0.0506	**0.111**

**Table 3:** The experiment result in BTC-e dataset.

		ANN	CNN	ARMA	SVR	Our model
*τ* = 10*s*	MSE	1.461	1.451	1.469	1.515	**1.413**
	*R* ^2^	0.0706	0.0770	0.0655	0.0368	**0.101**
*τ* = 20*s*	MSE	2.201	2.181	2.176	2.331	**2.143**
	*R* ^2^	0.0806	0.0890	0.0911	0.0265	**0.105**
*τ* = 40*s*	MSE	3.429	3.468	3.360	3.518	**3.342**
	*R* ^2^	0.0871	0.0767	0.105	0.0634	**0.110**
*τ* = 80*s*	MSE	6.197	7.257	**6.012**	6.269	6.238
	*R* ^2^	0.101	0.0630	**0.127**	0.0904	0.0948

As for *R*^2^, it is found that our method also outperforms all other baselines. When *τ* = 10*s*, the *R*^2^ in our method is 0.219, while it is 0.174 for ARMA. The improvement is more than 25%, which is significant. It is near 10% improvement when *τ* = 80*s*.

**Fig 2:**
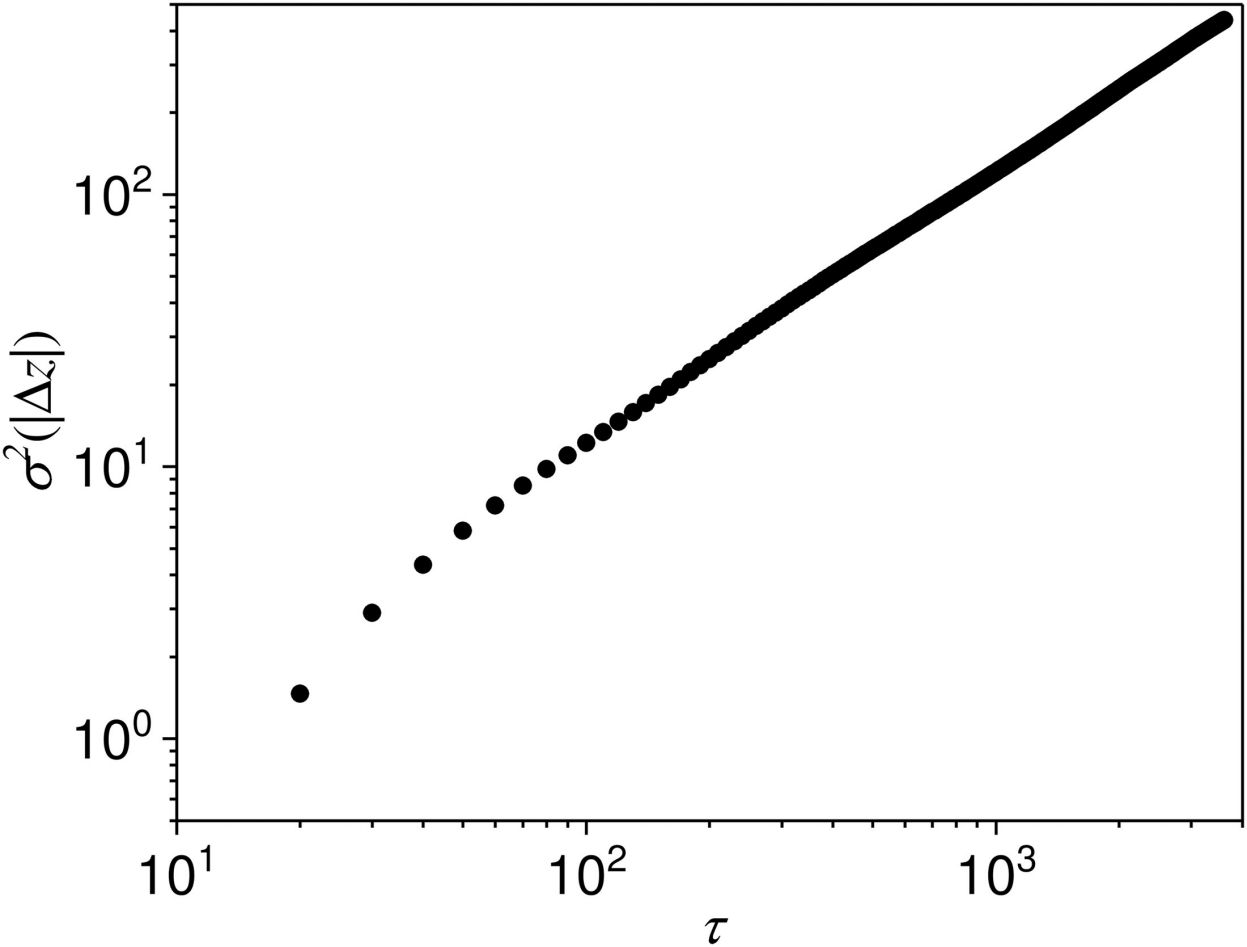
The variance of the change of price versus time interval *τ*.

On the other hand, we find that the experiment results on MSE increase as the time interval *τ* increase. Also, the experiment results on *R*^2^ decrease when time interval *τ* increases. It is because that the variance increases when time interval *τ* increases. Fig 2 shows the relationship between the variance of the change of price and time interval *τ*. The variance of the change of price is linear to the time interval *τ*. Thus, the predictability of the fluctuation decrease when time interval *τ* increase.

## Method

### Fluctuation prediction model

In this section, we will introduce our model, including the motivation, the framework of our model, the influence of order, the influence of the fluctuation at last moment and parameters update.

### Basic definition

There are some basis terms which need to be introduced.

- **Limit Order**. Limit order definition: https://www.investopedia.com/terms/l/limitorder.asp. A limit order is a type of order to purchase or sell a security at a specified price or better. For buying limit orders, the order will be executed only at the limit price or a lower one, while for selling limit orders, the order will be executed only at the limit price or a higher one. **Note: the order mentioned in this paper is the limit order**.
- **Order book**. Order book definition: https://www.investopedia.com/terms/o/order-book.asp. An order book is an electronic list of buying and selling orders for a specific security or financial instrument organized at a price level. An order book lists the number of shares being bid or offered at each price point.

**Fig 3:**
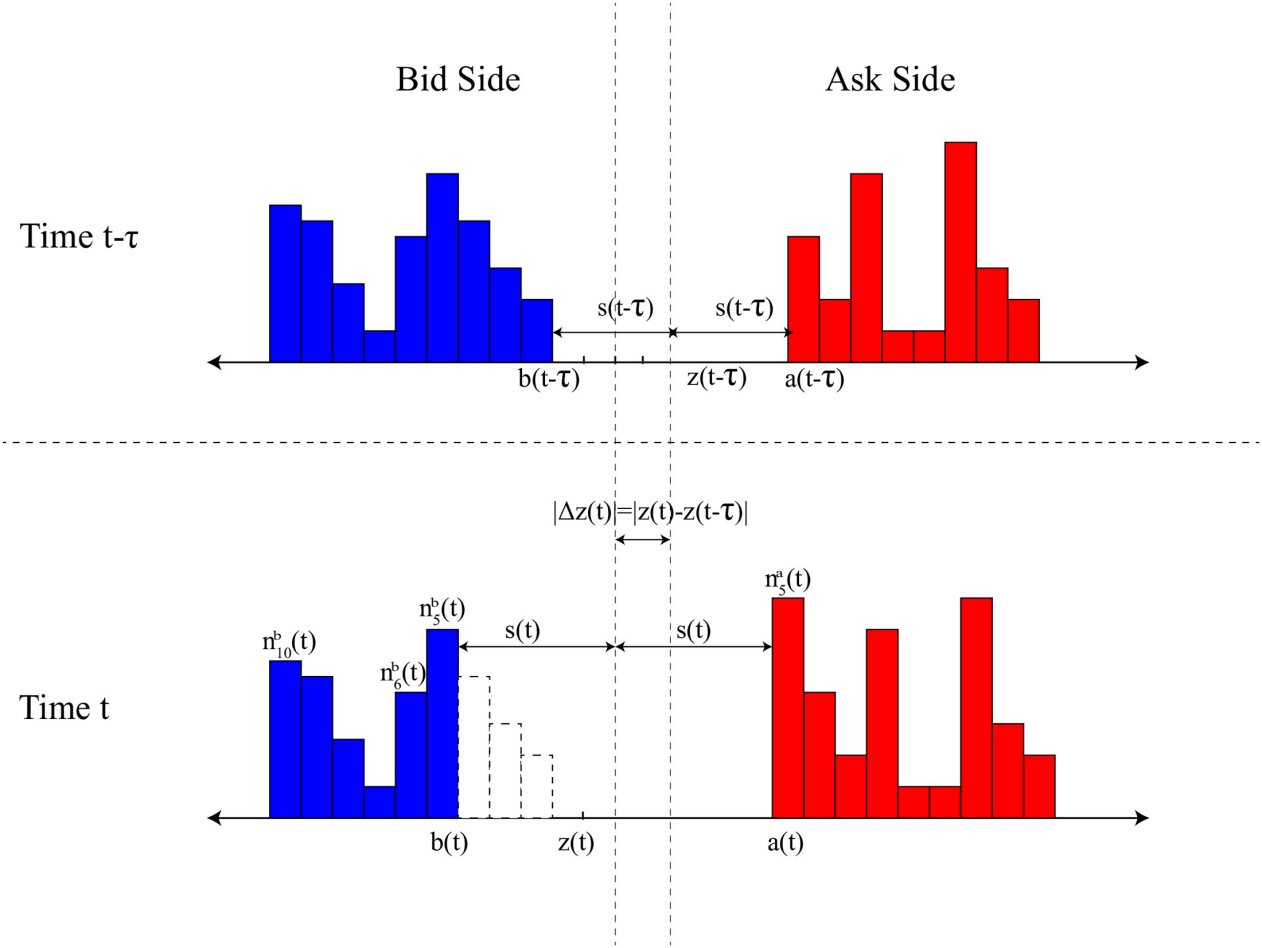
The change in order book from time *t* − *τ* to time *t*. The blue orders and red orders represent bid orders and ask orders, respectively.

Fig 3 demonstrates typical order book profiles at time *t* − *τ* and time *t*. The best ask *a*(*t*) (or best bid *b*(*t*)) is defined to be the lowest ask price (or the highest bid price) at time *t*. The midprice *z*(*t*) is calculated as $z\left(t\right)=\frac{a(t)+b(t)}{2}$. And the spread *s*(*t*) = *a*(*t*) − *b*(*t*), which represents the gap between best ask and best bid.

The blue orders and red orders represent bid order and ask order, respectively. And $n_{x}^{a}\left(t\right)$ and $n_{x}^{b}\left(t\right)$ represent the volume of ask order and bid order at time *t*, respectively. The relative position $x=\lceil \frac{|z_{x}-z\left(t\right)|}{\bar{z}}\rceil$ is the distances from price *z*_*x*_ to the midprice *z*(*t*), where *z*_*x*_ is the price of order and $\bar{z}$ is the price unit. Thus, the value $n_{x}^{a}\left(t\right)$ is the total volume of ask order from $z\left(t\right)+x*\bar{z}$ to $z\left(t\right)+\left(x+1\right)*\bar{z}$. The value $n_{x}^{b}\left(t\right)$ is the total volume of ask order from $z\left(t\right)-x*\bar{p}$ to $z\left(t\right)-\left(x+1\right)*\bar{z}$.

### Motivation

**Fig 4:**
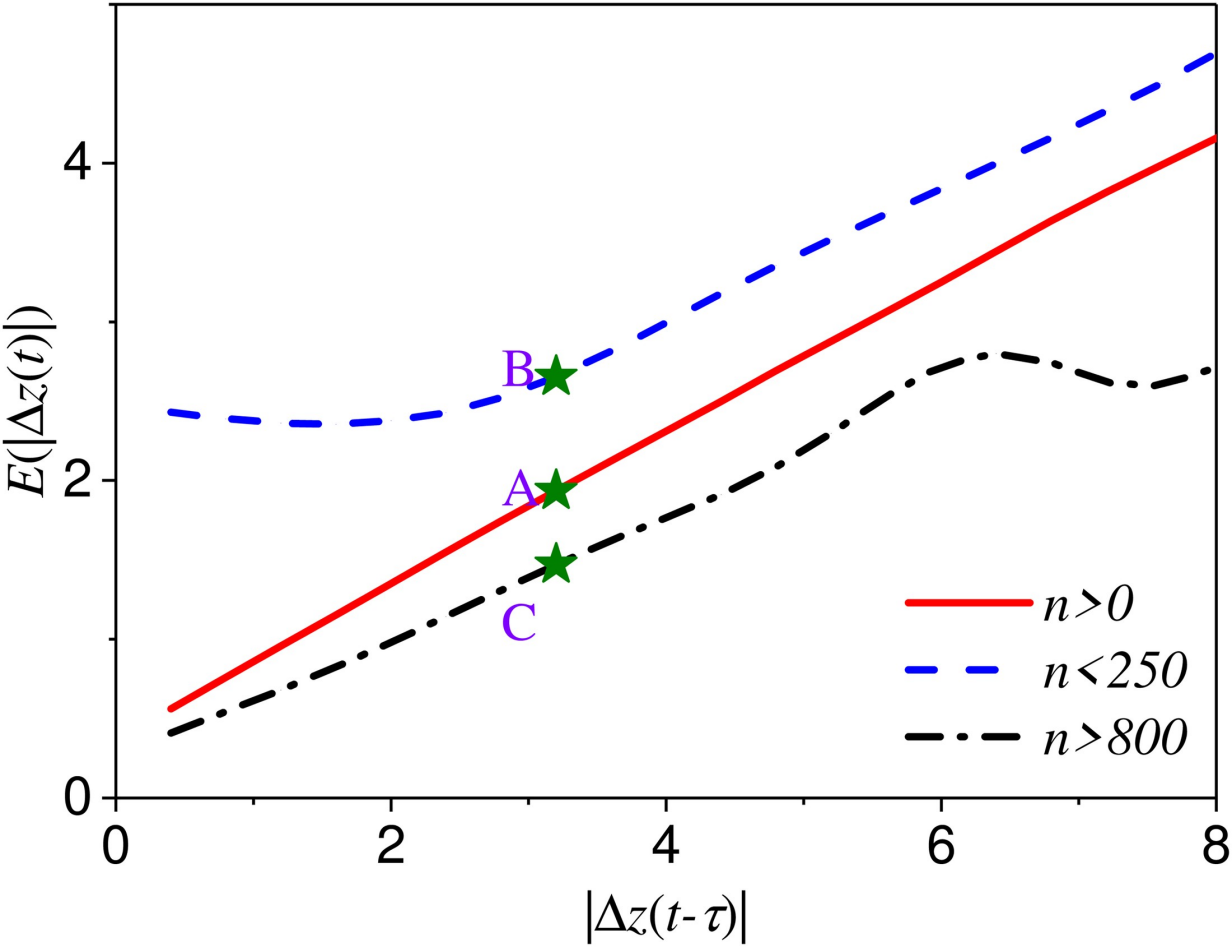
The relationship between the fluctuation of price in time *t* − *τ* and the fluctuation in time *t* for different order volumes.

Fig 4 shows the relationship between the fluctuation |Δ*z*(*t*)| at *t* and the fluctuation |Δ*z*(*t* − *τ*)| at *t* − *τ*. The red line is the expected value of prediction fluctuation |Δ*z*(*t*)| without considering the the volume of order. And the blue line and black line are the expected value of prediction fluctuation when *n*_0_ < 250 and *n*_0_ > 800 (*n*_0_ is the sum of order volume at best bid and best ask). The expected value of prediction fluctuation *E*(|Δ*z*(*t*)|) is obviously different when the fluctuation at last moment |Δ*z*(*t* − *τ*)| is the same. When *n*_0_ < 250, the fluctuation prediction is bigger than that *n*_0_ > 800. The redline is situated between the two. For example, when Δ*z*(*t* − *τ*) = 3, the expected value of fluctuation at next time *E*(|Δ*z*(*t*)|) = 1.3 for *n*_0_ > 800 (C point) and *E*(|Δ*z*(*t*)|) = 2.4 for *n*_0_ < 250 (A point). It seems that the smaller the order volume *n*_0_ is, the larger the fluctuation is. The fluctuation is related to the order volume *n*_0_.

Moreover, we consider the prediction value and the fluctuation |Δ*z*(*t* − *τ*)|. When the order information is given, the prediction value of fluctuation is

$$\begin{array}{c} f_{1}=\sum_{d}P(|\Delta z^{\prime }\left(t\right)|=d|\Delta z\left(t-\tau \right),n_{0})d. \end{array}$$ (5)

Only considering the price information, the prediction value is

$$\begin{array}{c} \begin{array}{cc} f_{2} & =\sum_{d}\sum_{n_{0}}P(|\Delta z^{\prime }\left(t\right)|=d|\Delta z\left(t-\tau \right),n_{0})d\\ & =\sum_{n_{0}}\sum_{d}P(|\Delta z^{\prime }\left(t\right)|=d|\Delta z\left(t-\tau \right),n_{0})d \end{array} \end{array}$$ (6)

Thus, this predition value *f*_1_ is the average value of *f*_2_ for different order volume. Obviously, the prediction value *f*_2_ would be more precision when the order information is given. Thus, the order information is conducive to fluctuation prediction.

To summarize, the order information is important and helpful for fluctuation prediction.

### Framework of model

In our model, the prediction of fluctuation aims to minimum the average squared difference between the prediction value and the actual value. Considering the order information, the loss function of fluctuation prediction *L* can be formation as:

$$\begin{array}{c} \begin{array}{cc} L= & \text{min}\sum_{t}[|\Delta z^{\prime }{\left(t\right)|-|\Delta z\left(t\right)|]}^{2}\\ = & \text{min}\sum_{t}[\sum_{d}^{DMAX}P(|\Delta z^{\prime }\left(t\right)|=d|H\left(t-\tau \right))d-{\left|\Delta z\left(t\right)|\right]}^{2}, \end{array} \end{array}$$ (7)

while |Δ*z*′(*t*)| is the value that our model predicts. The history information *H*(*t* − *τ*) = {Δ*z*(*t* − *τ*), *n*(*t* − *τ*)}, which represents the history data of order book and price. The value *DMAX* is the maximum value of price fluctuation, which is hyperparameters in our model. From Eq (7), it is significantly that the key for this problem is to calculate *P*(|Δ*z*′(*t*)| = *d*|*H*(*t* − *τ*)).

The value *P*(|Δ*z*′(*t*)| = *d*|*H*(*t* − *τ*)) is the probability of the change of price, which can be converted to the probability of the change of order. From the price perspective, the price changes from $z\left(t-\tau \right)=\frac{a(t-\tau )+b(t-\tau )}{2}$ to $z\left(t\right)=\frac{a(t)+b(t)}{2}$ (Fig 3). And the best bid(ask) price changes from *b*(*t* − *τ*)(*a*(*t* − *τ*)) to *b*(*t*)(*a*(*t*)). From the order perspective, the order with the price *z*_*x*_ ∈ [*b*(*t* − *τ*), *b*(*t*)] disappear when the best bid change from *b*(*t* − *τ*) to *b*(*t*). Thus, the change in price is actually equivalent to the change in the order volume.

Based on this observation, the prediction of fluctuation can be converted into the prediction of the order volume. The conditional probability *P*(|Δ*z*′(*t*)| = *d*|*H*(*t* − *τ*)) is calculated as

$$\begin{array}{c} \begin{array}{cc} & P(|\Delta z^{\prime }\left(t\right)|=d|H\left(t-\tau \right))\\ = & P(|a(t)-a(t-\tau )+b(t)-b(t-\tau )|=2d|H(t-\tau ))\\ = & \sum_{|i+j|=2d}P(a(t)-a(t-\tau )=i,b(t)-b(t-\tau )=j|H(t-\tau ))\\ = & \sum_{|i+j|=2d}P(n_{1}^{a}\left(t\right)=0,...,n_{s(t-\tau )+i-1}^{a}\left(t\right)=0,n_{s(t-\tau )+i}^{a}\left(t\right)>0,\\ & \;n_{1}^{b}\left(t\right)=0,...,n_{s(t-\tau )+j-1}^{b}\left(t\right)=0,n_{s(t-\tau )+j}^{b}\left(t\right)>0\left|H\left(t-\tau \right)\right) \end{array} \end{array}$$ (8)

To simplify our model, we assume that the changes of order at different positions are independent. Thus,

$$\begin{array}{c} \begin{array}{cc} & P(|\Delta z^{\prime }\left(t\right)|=d|H\left(t-\tau \right))\\ = & \sum_{|i+j|=2d}\prod_{k=0}^{s(t-\tau )+i-1}P(n_{k}^{a}\left(t\right)=0|H\left(t-\tau \right))P\left(n_{s(t-\tau )+i}^{a}\left(t\right)>0|H\left(t-\tau \right)\right)\\ & \;\prod_{k=0}^{s(t-\tau )+j-1}P(n_{k}^{b}\left(t\right)=0|H\left(t-\tau \right))P\left(n_{s(t-\tau )+j}^{b}\left(t\right)>0|H\left(t-\tau \right)\right) \end{array} \end{array}$$ (9)

Thus, how to predict the volume of order at next time is key for the prediction of fluctuation.

#### Influence of orders

The change of an order volume is resulted from two operation: order placement and order cancellation.

- **Order placement**. The order placement at time *t* denotes as $\Delta n_{i}^{OP}\left(t\right)$. The previous study shows that the order placement is related the order price.
- **Order cancellation**. The order cancelled at time *t* denotes as $\Delta n_{i}^{OC}\left(t\right)$. We assume that every unit order is cancelled with the same probability *q*_*i*_. Based on this assumption, the process of cancellation is similar to the box picking problem: there are $\frac{n_{i}}{c}$ ball in the box at time *t* − *τ*, while *c* is the minimum unit of order. And the balls are picked with the same probability *q*_*i*_ independently. Therefore the probability $P(\Delta n_{i}^{OC}\left(t-\tau \right)=m|H\left(t-\tau \right))$ can be calculated as
  $$\begin{array}{c} \begin{array}{cc} & P(\Delta n_{i}^{OC}\left(t-\tau \right)=m|H\left(t-\tau \right))\\ = & C\left(\frac{n_{i}\left(t-\tau \right)}{c},\frac{n_{i}\left(t-\tau \right)-m}{c}\right){\left(1-q_{i}\right)}^{\frac{n_{i}\left(t-\tau \right)-m}{c}}q_{i}^{\frac{m}{c}}\\ \approx & \frac{{\left[n_{i}\left(t-\tau \right)\left(1-\frac{q_{i}}{c}\right)\right]}^{\frac{n_{i}\left(t-\tau \right)-m}{c}}}{(\frac{n_{i}\left(t-\tau \right)-m}{c})!}e^{-n_{i}\left(t-\tau \right)\left(1-\frac{q_{i}}{c}\right)} \end{array} \end{array}$$ (10)
  The approximation in Eq (10) is because ${\text{lim}}_{n\to \infty ,p\to 0}C_{n}^{k}p^{k}{\left(1-p\right)}^{n-k}\approx \frac{{\left(np\right)}^{k}}{k!}e^{-np}$. When *m* = *n*_*i*_(*t* − *τ*), the conditional probability $P\left(\Delta n_{i}^{OC}\left(t-\tau \right)=m|H\left(t-\tau \right)\right)\approx e^{-n_{i}\left(t-\tau \right)\left(1-\frac{q_{i}}{c}\right)}$.

Based on the analysis above, the probability *P*(*n*_*i*_(*t*) = 0|*H*(*t* − *τ*)) can be calculated as:

$$\begin{array}{c} \begin{array}{cc} & P(n_{i}\left(t\right)=0|H\left(t-\tau \right))\\ = & P(n_{i}\left(t-\tau \right)+\Delta n_{i}^{OP}\left(t-\tau \right)-\Delta n_{i}^{OC}\left(t-\tau \right)=0|H\left(t-\tau \right))\\ = & P\left(\Delta n_{i}^{OP}\left(t-\tau \right)=0|H\left(t-\tau \right)\right)P\left(\Delta n_{i}^{OC}\left(t-\tau \right)=n_{i}\left(t-\tau \right)|H\left(t-\tau \right)\right)\\ = & P\left(\Delta n_{i}^{OP}\left(t-\tau \right)=0|H\left(t-\tau \right)\right)e^{-n_{i}\left(t-\tau \right)\left(1-\frac{q_{i}}{c}\right)} \end{array} \end{array}$$ (11)

Eq (11) show the relationship between the probability *P*(*n*_*i*_(*t*) = 0|*H*(*t* − *τ*)) and the order volume *n*_*i*_(*t* − *τ*). Thus, we set the formation of the probability *P*(*n*_*i*_(*t*) = 0|*H*(*t* − *τ*)) as

$$\begin{array}{c} \begin{array}{cc} & P(n_{i}\left(t\right)=0|H\left(t-\tau \right))\\ = & w_{i}\left(1\right)e^{w_{i}\left(2\right)n_{i}\left(t-\tau \right)+w_{i}\left(3\right)n_{i}\left(t-\tau \right)q_{i}}+w_{i}\left(4\right). \end{array} \end{array}$$ (12)

**Fig 5:**
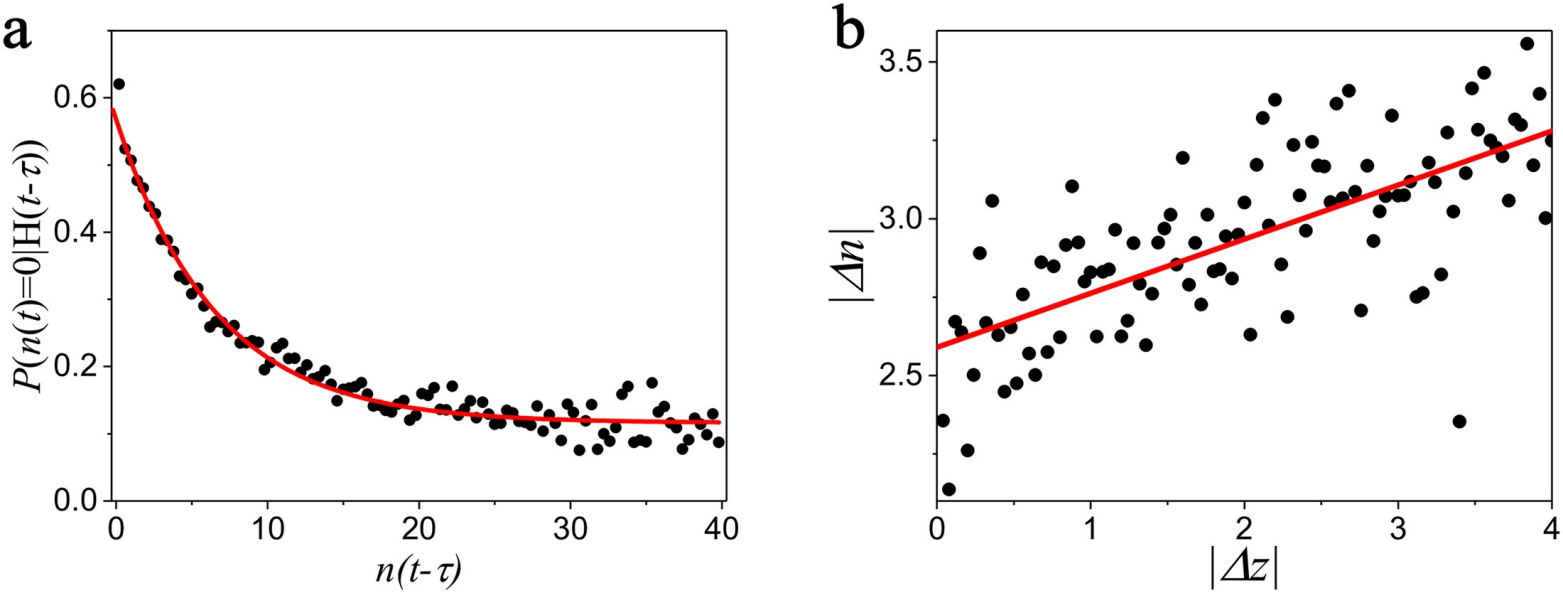
**a**. The relationship between the conditional probability *P*(*n*(*t*) = 0|*H*(*t* − *τ*)) and the order volume *n*(*t* − *τ*). The red line is the fitting line. **b**. The relationship between the absolute value of the change of order volume |Δ*n*| versus the fluctuation |Δ*z*|.

To verify whether our model is correct, we calculate the conditional probability *P*(*n*(*t*) = 0|*H*(*t* − *τ*)) for different order volume *n*(*t* − *τ*) in Bitcoin transaction platform. We find the conditional probability *P*(*n*(*t*) = 0|*H*(*t* − *τ*)) decreases as the volume of order *n*(*t* − *τ*) increases in Fig 5. It is found that $P\left(n\left(t\right)=0|H\left(t-\tau \right)\right)=w_{i}\left(1\right)e^{w_{i}\left(2\right)n\left(t-\tau \right)}+w_{i}\left(3\right)$ fit the data well, which means that our model capture the relationship between *P*(*n*_*i*_(*t*) = 0|*H*(*t* − *τ*)) and *n*_*i*_(*t* − *τ*).

#### Influence of the fluctuation at last moment

In addition, we need to determine the the relationship between rate of cancellation *q*_*i*_ and the fluctuation |Δ*z*(*t*)|. Fig 5b shows the relationship between |Δ*n*| and |Δ*z*|. It is found that the change of order volume |Δ*n*| increases as the fluctuation |Δ*z*| increases, implying that *q*_*i*_ is related to Δ*z*(*t*). To simplify our model, we set *q*_*i*_ ≈ *k*|Δ*z*(*t*)|. However, |Δ*z*(*t*)| is unknown when we predict the fluctuation at time *t* − *τ*. So we replace |Δ*z*(*t*)| with the average fluctuation before ∑_*j*_|Δ*z*(*t* − *j*)|. Thus, Eq (12) can be rewrite as

$$\begin{array}{c} \begin{array}{cc} & P(n_{i}\left(t\right)=0|H\left(t-\tau \right))\\ = & w_{i}\left(1\right)e^{w_{i}\left(2\right)n_{i}\left(t-\tau \right)+w_{i}\left(3\right)n_{i}\left(t-\tau \right)\sum_{j}\left|\Delta z\left(t-j\right)\right|}+w_{i}\left(4\right). \end{array} \end{array}$$ (13)

#### Parameters update

In our model the parameters *w*_*i*_(1), *w*_*i*_(2), *w*_*i*_(3), *w*_*i*_(4), *w*_*i*_(5) are need to form the train data. Using the stochastic gradient descent method, the parameter *w*_*i*_(2)is updated as

$$\begin{array}{cc} w_{i}\left(2\right) & =w_{i}\left(2\right)+\eta \frac{\partial L}{\partial w_{i}\left(2\right)}\\ & =w_{i}\left(2\right)+\eta \frac{\partial L}{\partial P(n_{i}\left(t\right)=0|H\left(t-\tau \right))}\frac{\partial P(n_{i}\left(t\right)=0|H\left(t-\tau \right))}{\partial w_{i}\left(2\right)}. \end{array}$$ (14)

But we find that the distribution of *n*_*i*_(*t* − *τ*) is fat-tail, which means that the maximum value of *n*_*i*_(*t*) is many times of the minimum value of *n*_*i*_(*t*). In the Bitcoin transaction platform, we find that the maximum value of *n*_*i*_(*t*) is more than 100 times the average value of *n*_*i*_(*t*). The extreme value of order volume would result in the parameter *w*_*i*_(2) fluctuate violently.

To solve this problem, we proposed a two-step method to update parameters(algorithm 1). At first, we calculate the conditional probability *P*(*n*_*i*_(*t*) = 0|*H*(*t* − *τ*)) using the stochastic gradient descent method [45]. Secondly, we use Eq (13) to fit *P*(*n*_*i*_(*t*) = 0|*H*(*t* − *τ*)). The difference between our method and the stochastic gradient descent method is that we replace derivation with fitting.

**Algorithm 1**: Learning the conditional probability *P*(*n*(*t*) = 0|*H*(*t* − *τ*))

  **Input**: The train dataset;

  **Output**: The probability *P*(*n*_*i*_(*t*) = 0|*H*(*t* − *τ*));

1 //ITER: Number of iterations; T: size of train dataset; DMAX: maximum price movement; LEN: depth of order book

2 **for** *i* = 1 to *ITER* **do**

3   // Train

4   **for** *t* = 1 to *T* **do**

5    **for** *d* = 1 to *DMAX* **do**

6     cal *P*(|Δ*z*′(*t*)| = *d*|*H*(*t* − *τ*)) using Eq (9)

7    **end**

8    1. calculate prediction value |Δ*z*′(*t*)|

9    2. calculate mean-square-error *MSE* = (|Δ*z*′(*t*)| − |Δ*z*(*t*)|)^2^

10    3. update conditional probability *P*(*n*(*t*) = 0|*H*(*t* − *τ*))

11   **end**

12   // Fit probability

13   **for** *k* = 1 to *LEN* **do**

14    Fit *P*(*n*_*k*_(*t*) = 0|*H*(*t* − *τ*)) using Eq (12)

15   **end**

16 **end**

17 Return *P*(*n*_*i*_(*t*) = 0|*H*(*t* − *τ*))

## Discussion

In this paper, there are five fluctuation prediction methods. The baseline models are the end-to-end models, which ignore the relationship between fluctuation and order. Comparing with the baseline models, we convert the prediction of price fluctuation into the prediction of order volume. Our model explicitly builds the relationship between the fluctuation and the order book. It uses more prior knowledge about order books and price. This may be the reason why our model performs better than the four baseline models.

High-frequency trading is the operation of seeking profits in the short-term market changes, which is usually carried out by computer programs. Dozens of orders can be placed in a second, and these orders will have an impact on the price. The study of the impact of order volume changes on short-term price, allows us to monitor high-frequency trading behaviour. However, the prediction of price direction is not within the scope of our study, because the price rise and fall direction of the actual market is not random distribution. The accuracy of predicting price trends (rise or fall) is out of this research scope. In the future, the prediction of price trends will be future work. Our method is not only suitable for the bitcoin market, but also for the order-driven market, for example the stock market which has a lot of high-frequency transactions.

## Conclusion

In this paper, we focus on the fluctuation prediction. We find that the price would move when the volume of order changes. Based on this observation, the prediction of price fluctuation can be converted into the order volume prediction. Modeling the trader’s behaviors—order placement and order cancellation, we conduct an order book based fluctuation prediction model. The developed model performs better on the OKCoin and BTC-e datasets. But there is a limitation on our model—the time lag that our model can predict is small. We try to solve this problem in the future work.
